# Atlas of lesion locations and postsurgical seizure freedom in focal cortical dysplasia: A MELD study

**DOI:** 10.1111/epi.17130

**Published:** 2021-11-29

**Authors:** Konrad Wagstyl, Kirstie Whitaker, Armin Raznahan, Jakob Seidlitz, Petra E. Vértes, Stephen Foldes, Zachary Humphreys, Wenhan Hu, Jiajie Mo, Marcus Likeman, Shirin Davies, Matteo Lenge, Nathan T. Cohen, Yingying Tang, Shan Wang, Mathilde Ripart, Aswin Chari, Martin Tisdall, Nuria Bargallo, Estefanía Conde‐Blanco, Jose Carlos Pariente, Saül Pascual‐Diaz, Ignacio Delgado‐Martínez, Carmen Pérez‐Enríquez, Ilaria Lagorio, Eugenio Abela, Nandini Mullatti, Jonathan O'Muircheartaigh, Katy Vecchiato, Yawu Liu, Maria Caligiuri, Ben Sinclair, Lucy Vivash, Anna Willard, Jothy Kandasamy, Ailsa McLellan, Drahoslav Sokol, Mira Semmelroch, Ane Kloster, Giske Opheim, Clarissa Yasuda, Kai Zhang, Khalid Hamandi, Carmen Barba, Renzo Guerrini, William Davis Gaillard, Xiaozhen You, Irene Wang, Sofía González‐Ortiz, Mariasavina Severino, Pasquale Striano, Domenico Tortora, Reetta Kalviainen, Antonio Gambardella, Angelo Labate, Patricia Desmond, Elaine Lui, Terry O'Brien, Jay Shetty, Graeme Jackson, John S. Duncan, Gavin P. Winston, Lars Pinborg, Fernando Cendes, Judith Helen Cross, Torsten Baldeweg, Sophie Adler

**Affiliations:** ^1^ Wellcome Centre for Human Neuroimaging London UK; ^2^ Alan Turing Institute London UK; ^3^ Developmental Neurogenomics National Institute of Mental Health Bethesda Maryland USA; ^4^ Department of Child and Adolescent Psychiatry and Behavioral Science Children’s Hospital of Philadelphia Philadelphia Pennsylvania USA; ^5^ Department of Psychiatry University of Pennsylvania Philadelphia Pennsylvania USA; ^6^ Department of Psychiatry, Behavioural and Clinical Neuroscience Institute University of Cambridge Cambridge UK; ^7^ Barrow Neurological Institute at Phoenix Children's Hospital Phoenix Arizona USA; ^8^ Department of Neurosurgery Beijing Tiantan Hospital Capital Medical University Beijing China; ^9^ Bristol Royal Hospital for Children Bristol UK; ^10^ Cardiff University Brain Research Imaging Centre School of Psychology Cardiff UK; ^11^ Welsh Epilepsy Unit Cardiff and Vale University Health Board University Hospital of Wales Cardiff UK; ^12^ Neuroscience Department Meyer Children's Hospital–University of Florence Florence Italy; ^13^ Center for Neuroscience Children's National Hospital Washington District of Columbia USA; ^14^ Department of Neurology West China Hospital of Sichuan University Chengdu China; ^15^ Epilepsy Center Neurological Institute Cleveland Clinic Cleveland Ohio USA; ^16^ Epilepsy Center Department of Neurology Epilepsy Center Second Affiliated Hospital School of Medicine Zhejiang University Hangzhou China; ^17^ University College London Great Ormond Street Institute for Child Health London UK; ^18^ Great Ormond Street Hospital National Health Service Foundation Trust UK; ^19^ Hospital Clinic of Barcelona Barcelona Spain; ^20^ Magnetic Resonance Core Image Facility August Pi i Sunyer Biomedical Research Institute Spain; ^21^ Hospital of the Sea Barceloneta Promenade Barcelona Spain; ^22^ Giannina Gaslini Institute, Scientific Institute for Research and Health Care Genova Italy; ^23^ Center for Neuropsychiatry and Intellectual Disability Aargau Psychiatric Services Windisch Switzerland; ^24^ Institute of Psychiatry, Psychology, and Neuroscience London UK; ^25^ Department of Neurology University of Eastern Finland Kuopio Finland; ^26^ Institute of Neurology Department of Medical and Surgical Sciences Magna Graecia University of Catanzaro Catanzaro Italy; ^27^ Department of Neuroscience Central Clinical School Monash University Melbourne Victoria Australia; ^28^ Department of Neurology Alfred Hospital Melbourne Victoria Australia; ^29^ Royal Hospital for Children and Young People Little France Crescent Edinburgh UK; ^30^ Florey Institute of Neuroscience and Mental Health Heidelberg Victoria Australia; ^31^ Neurobiology Research Unit and Epilepsy Clinic Department of Neurology Copenhagen University Hospital Rigshopsitalet Copenhagen Denmark; ^32^ Department of Neurology University of Campinas Campinas Brazil; ^33^ Kuopio Epilepsy Center Neurocenter Kuopio University Hospital Kuopio Finland; ^34^ Department of Radiology Royal Melbourne Hospital Parkville Victoria Australia; ^35^ University College London Queen Square Institute of Neurology London UK; ^36^ Division of Neurology Department of Medicine Queen’s University Kingston Ontario Canada

**Keywords:** drug‐resistant epilepsy, focal cortical dysplasia, lesions, MRI, neurosurgery

## Abstract

**Objective:**

Drug‐resistant focal epilepsy is often caused by focal cortical dysplasias (FCDs). The distribution of these lesions across the cerebral cortex and the impact of lesion location on clinical presentation and surgical outcome are largely unknown. We created a neuroimaging cohort of patients with individually mapped FCDs to determine factors associated with lesion location and predictors of postsurgical outcome.

**Methods:**

The MELD (Multi‐centre Epilepsy Lesion Detection) project collated a retrospective cohort of 580 patients with epilepsy attributed to FCD from 20 epilepsy centers worldwide. Magnetic resonance imaging‐based maps of individual FCDs with accompanying demographic, clinical, and surgical information were collected. We mapped the distribution of FCDs, examined for associations between clinical factors and lesion location, and developed a predictive model of postsurgical seizure freedom.

**Results:**

FCDs were nonuniformly distributed, concentrating in the superior frontal sulcus, frontal pole, and temporal pole. Epilepsy onset was typically before the age of 10 years. Earlier epilepsy onset was associated with lesions in primary sensory areas, whereas later epilepsy onset was associated with lesions in association cortices. Lesions in temporal and occipital lobes tended to be larger than frontal lobe lesions. Seizure freedom rates varied with FCD location, from around 30% in visual, motor, and premotor areas to 75% in superior temporal and frontal gyri. The predictive model of postsurgical seizure freedom had a positive predictive value of 70% and negative predictive value of 61%.

**Significance:**

FCD location is an important determinant of its size, the age at epilepsy onset, and the likelihood of seizure freedom postsurgery. Our atlas of lesion locations can be used to guide the radiological search for subtle lesions in individual patients. Our atlas of regional seizure freedom rates and associated predictive model can be used to estimate individual likelihoods of postsurgical seizure freedom. Data‐driven atlases and predictive models are essential for evidence‐based, precision medicine and risk counseling in epilepsy.

**Key Points**

- Atlas of focal lesions reveals nonuniform distribution, with lesions clustered around superior frontal sulcus, frontal pole, and temporal pole
- Earlier epilepsy onset was associated with lesions in primary sensory areas, whereas later epilepsy onset was associated with lesions in association cortices
- Seizure freedom rates varied with FCD location, being approximately 30% in visual, motor, and premotor areas and 75% in superior temporal and frontal gyri
- The predictive model of postsurgical seizure freedom, including lesional overlap with eloquent cortex, had a positive predictive value of 70% and negative predictive value of 61%

## 1 INTRODUCTION

Epilepsy is one of the most common neurological conditions, with a lifetime risk of one in 26.^1^ Focal cortical dysplasia (FCD) is a malformation of cortical development and common cause of drug‐resistant epilepsy.^2^, ^3^ In many patients, FCD is amenable to surgical resection, with reported long‐term seizure freedom rates of 69%.^4^

FCDs can be categorized into distinct histopathological subtypes.^2^ FCD Type I is characterized by cortical dyslamination. FCD Type II is characterized by dysmorphic neurons and dyslamination, and is subdivided into IIA and IIB, with the latter having balloon cells. FCD Type III is associated with another principal lesion, for example, hippocampal sclerosis.

FCDs can occur anywhere in the cerebral cortex, but different histopathological subtypes show some lobar specificity.^2^, ^3^ FCD Type II lesions are more frequently found in the frontal lobe, whereas FCD Type I and III are more frequently located in the temporal lobe. To date, most studies analyzing localization have used coarse lobar categorizations^4^ or have been limited by small sample sizes.^5^ Despite anatomical mapping of lesions being available using presurgical magnetic resonance imaging (MRI)^6^, ^7^, ^8^ and the emergence of large collaborative neuroimaging studies,^9^ these techniques have not been used to map the topography of FCDs.

The gold standard treatment for drug‐resistant focal epilepsy is surgical resection.^10^ However, a significant proportion of patients (31% of FCD Type II and 42% of FCD Type I)^4^ continue to have seizures postoperatively. Identifying factors relating to seizure freedom is important. Some can be modified to improve patients’ clinical and surgical treatment. Others can be incorporated into machine‐learning models to produce patient‐specific predictions of seizure freedom for use in clinical planning and risk counseling. Across all focal epilepsies, duration of epilepsy, age at surgery, lesion lobe, and histopathological diagnosis are predictors of postsurgical freedom.^4^ Within FCD, the most consistent predictive factors include complete resection of the FCD,^11^, ^12^ temporal resections,^13^, ^14^ having an MRI‐visible lesion,^15^ and the underlying histopathology being FCD Type II.^4^ However, the relationship between precise lesion location and seizure freedom is unknown.

Detailed spatial mapping of FCD lesions would broaden our understanding of this disease, enabling linkage of a patient's lesion location to presurgical clinical information. To this end, we created the Multi‐centre Epilepsy Lesion Detection (MELD) project to collate a large neuroimaging cohort of patients, including MRI lesion maps with demographic, clinical, and surgical variables. We aimed to (1) map the topographic distribution of epileptogenic FCDs across the cerebral cortex, (2) identify clinical factors associated with lesion location, and (3) establish predictors of postsurgical seizure freedom.

## 2 MATERIALS AND METHODS

### 2.1 MELD project consortium

We established the MELD project (https://meldproject.github.io/), involving 20 research centers across five continents. Each center received approval from their local institutional review board or ethics committee.

### 2.2 Participants

Patients older than 3 years were included if they had a three‐dimensional (3D) T1‐weighted MRI brain scan (1.5 T or 3 T) and a radiological diagnosis of FCD or were MRI‐negative with histopathological confirmation of FCD. Participants were excluded if they had previous surgeries, large structural abnormalities in addition to the FCD, or T1 scans with gadolinium enhancement.

### 2.3 Site‐level data collection and postprocessing

#### 2.3.1 MRI data collection and postprocessing

Preoperative T1‐weighted MRI scans were collected at the 20 participating centers for all participants, and cortical surfaces were reconstructed using FreeSurfer (v5.3 or v6).^16^

#### 2.3.2 FCD lesion masking

FCD lesions were delineated on the T1‐weighted MRI scans by a neuroradiologist or experienced epilepsy researcher at each site.^17^ For MRI‐negative, histopathologically confirmed patients, the resection cavity from the postoperative scan was used to help segment the FCD. Volumetric lesion masks were mapped to cortical reconstructions, and small defects were filled using a dilation‐erosion algorithm. Patients’ lesions were registered to a bilaterally symmetrical template surface, fsaverage_sym. Lesion size was calculated as the percentage of lesional vertices in that hemisphere.

#### 2.3.3 Participant demographics

The following data were collected for all patients: age at MRI scan, sex, age at epilepsy onset, duration of epilepsy (time from age at epilepsy onset to age at MRI scan), ever reported MRI‐negative, histopathological diagnosis (International League Against Epilepsy three‐tiered classification system),^2^ seizure freedom (Engel class I, minimum follow‐up time of 1 year), and follow‐up time in operated patients. Deidentified demographic information and lesion masks were shared with the study coordinators for multicenter analysis.

### 2.4 Location of FCDs in the cerebral cortex

Lesion masks from all patients were overlaid on the left hemisphere of the template surface to visualize the distribution of lesions for all FCDs, as well as for histopathological subtypes (see Materials and Methods Section 2.5 for tests of interhemispheric asymmetry). Additional surface‐based lesion maps for left and right hemispheres were generated for the whole cohort, patients who had, and those who had not undergone resective surgery.

Lobar categorizations of lesion location were created based on the lobe that contained the most lesional vertices. For statistical comparisons of lobar location, lesions primarily located in the smallest lobes in the parcellation (cingulate and insula) were assigned to a second lobe (e.g., frontal), which the lesion mask also overlapped. A mask of eloquent cortex was created including the following cortical areas bilaterally from the Desikan–Killiany atlas: precentral (motor), pericalcarine, lateral occipital, cuneus, and lingual labels (all visual cortical areas). Additionally, the pars opercularis, pars triangularis, and transverse temporal labels (language areas) were included on the left hemisphere.

For the creation of a 3D lesion likelihood atlas, aggregated surface‐based lesion map values were normalized to between 0 and 1, where the location with the highest number of FCDs had a value of 1. This lesion likelihood atlas was mapped back to the template MRI volume for use as a clinical aid in guiding radiological diagnosis.

Bootstrap reproducibility was used to assess the consistency of the overall spatial distribution of FCDs. FCD frequency maps from subsets of 20–250 patients were correlated with the map from a subset of 250 patients. This process was randomized and repeated 1000 times. Unstable maps had low correlations and indicated that the sample size was too small. A predictive learning curve was fitted to the *R* values of different subcohort sizes and interpolated to determine the sample size required for a consistent spatial distribution in the lesion pattern.^18^

### 2.5 Factors associated with lesion distribution

We trained logistic regression models to test for associations between lesion location and clinical data (Supplementary Figure S1). The models were fitted to assess which preoperative factors are associated with the presence or absence of lesions at a particular vertex (a point on the cortical surface). The following preoperative factors were included: sex, age at epilepsy onset, duration of epilepsy, ever reported MRI‐negative, lesion size, and lesion hemisphere. Due to collinearity with duration of epilepsy (*r* = 0.76), age at MRI scan was tested in a separate model excluding duration. Regression coefficients were tested for significance against those calculated on 1000 randomly permuted cohorts. Factors were deemed significant if their coefficient was <2.5% or >97.5% of the coefficients from permuted cohorts. Approximately 5% of vertices would be significant by chance. A given factor was considered significantly related to lesion location if the number of significant vertices exceeded the 100 − (5/*n*th) percentile number of significant vertices from the permuted cohorts, where *n* was the number of factors being tested.

Post hoc analysis of significant factors included testing for similarity between the surface‐based map of age at epilepsy onset and a potential explanatory variable, the principal axis of cortical developmental organization from primary to association areas.^19^ To account for spatial autocorrelation, statistical significance was established by comparing correlations with those from 1000 spherically rotated maps.^20^

### 2.6 Factors associated with seizure freedom

Using the cohort of operated FCD patients with seizure outcome, we calculated the proportion of patients who were seizure‐free with lesions at each vertex. To assess the association of lesion location with postsurgical seizure freedom, along with other clinical factors, three logistic regression models were fitted. The first was solely based on lesion location, predicting seizure freedom at each cortical vertex based on which patients had lesions overlapping that vertex. The second was fitted to predict seizure freedom using the following presurgically available factors: duration of epilepsy, age at epilepsy onset, MRI‐negative status, scanner, lesion overlap with eloquent cortex, and lesion size. Due to collinearity, age at MRI scan was tested in a separate model excluding duration. A third model included the postoperatively determined histopathological FCD subtype and an interaction between lesion size and histopathological subtype. We calculated the predicted percentage likelihood of seizure freedom from each model for each patient. Statistical significance for these regression models was established using the same permutation procedures as in Materials and Methods Section 5.

To assess the predictive value of presurgical factors (Model 2) in determining postsurgical seizure freedom, we carried out 10‐fold cross‐validation. Sensitivity, specificity, positive predictive value, and negative predictive value were calculated.

### 2.7 Comprehensive search for interrelationships between demographic, lesional, and surgical variables

Relationships between demographic, lesion, and surgical variables were systematically analyzed (Figure 4B). Heavily skewed variables were normalized using a Box–Cox transform. The Benjamini–Hochberg procedure was used to control the false discovery rate when conducting multiple comparisons, with *α* set to .05.^21^

### 2.8 Code

All analyses were performed in Python using the following packages: NumPy, scikit‐learn, scipy, pandas, nibabel, matplotlib, seaborn, and PtitPrince. All protocols and code for MELD preprocessing and POOL (Prediction of Outcome & Location) are available to download from protocols.io/researchers/meld‐project and www.github.com/MELDProject.

### 2.9 Data availability

Lesion maps for the whole cohort are freely available to download from the MELD github (www.github.com/MELDProject). Requests can be made for access to the full MELD dataset.

## 3 RESULTS

### 3.1 Participant demographics

Data were collected from 580 FCD patients. Demographic information is available in Table 1. Histopathological diagnosis was available in 380 patients (66%; Table 2). Postsurgical outcome data with 1‐year minimum follow‐up were available in 275 patients; 65% were seizure‐free (Engel Class I) at last follow‐up (median follow‐up =2 years). Seizure freedom rates across histopathological subtypes are presented in Table 2. Although FCD Type I had a lower mean seizure freedom (55%) than FCD Type IIA (69%) and FCD Type IIB (66%), there was no significant difference in outcome according to histopathological subtype.

**TABLE 1:** Demographics table

Characteristic	FCD patients, *n* = 580
Age at scan, median years (IQR)	19.0 (11.0–31.3)
Sex, female:male	281:298
Age at onset, median years (IQR)	6.0 (2.5–12.0)
Ever reported MRI‐negative	188/580 (32%)
Duration of epilepsy, median years (IQR)	10.4 (4.9–19.0)
Surgery performed	423/580 (73%)
Histopathology available	380/580 (66%)
Outcome available	275/580 (47%)
Follow‐up time, median years (IQR)	2.0 (1.3–3.4)

Abbreviations: FCD, focal cortical dysplasia; IQR, interquartile range; MRI, magnetic resonance imaging.

**TABLE 2:** Histopathology and surgical outcome

FCD subtype	Histopathology, *n* (%)	Seizure‐free, *n* (%)	Ever reported MRI‐negative, *n* (%)
All	380 (100%)	165/252 (65%)	135/380 (36%)
FCD Type I	42 (11%)	17/31 (55%)	17/42 (40%)
FCD Type IIA	118 (31%)	58/84 (69%)	55/118 (47%)
FCD Type IIB	199 (52%)	80/121 (66%)	55/199 (28%)
FCD Type III	21 (6%)	10/16 (62%)	8/21 (38%)

Note

Histopathology = histopathological diagnosis; Histopathology % = percentage of patients with particular histopathological diagnosis out of the total number of patients with histopathology; Seizure‐free = number of patients with a particular histopathological diagnosis who were seizure‐free out of the total number of patients with FCD subtype and outcome data available; *n* = number of patients.

Abbreviations: FCD, focal cortical dysplasia; MRI, magnetic resonance imaging.

### 3.2 Location of FCDs in the cerebral cortex

**FIGURE 1:**
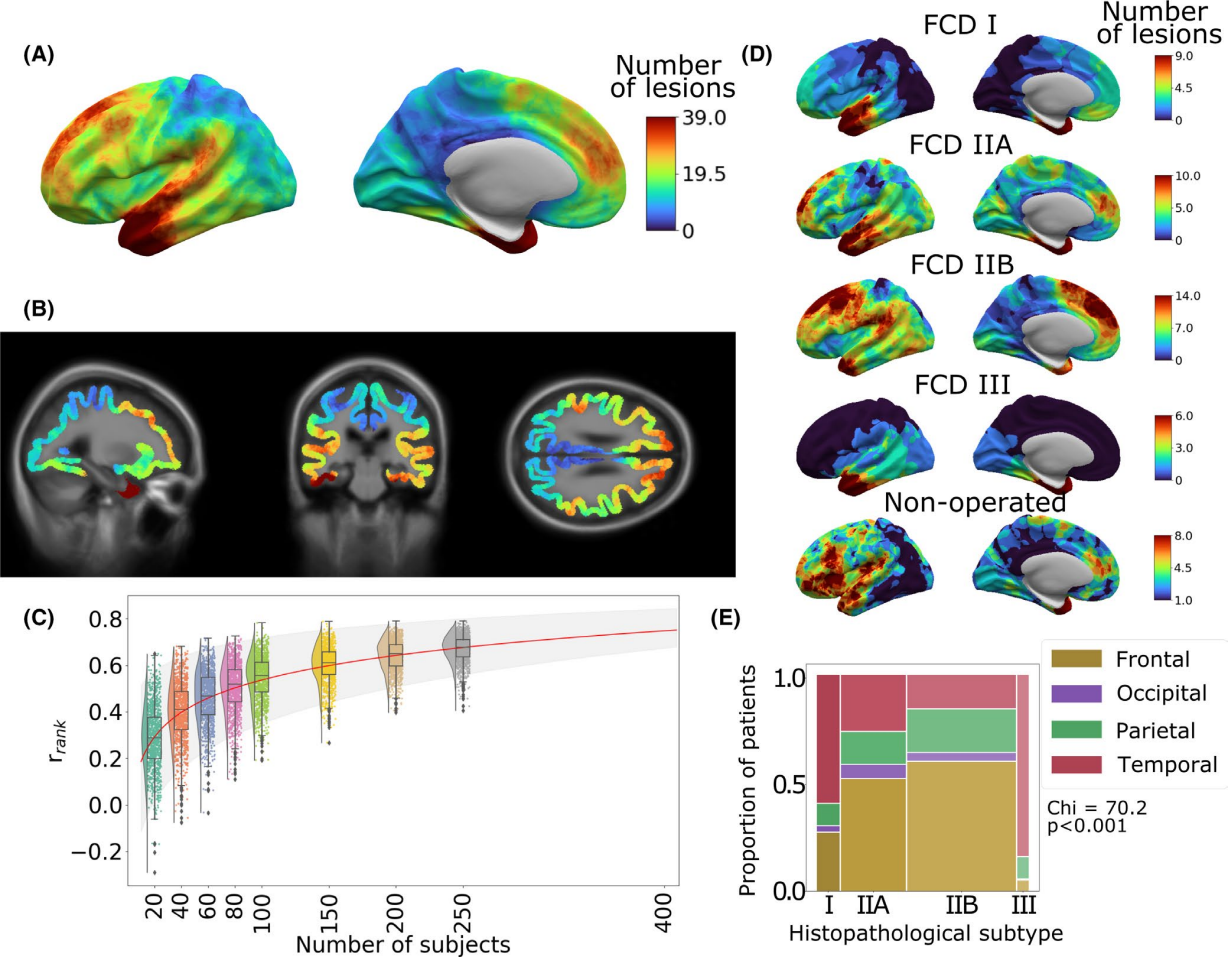
Distribution of focal cortical dysplasia (FCD) lesions across the cerebral cortex. (A) All FCD lesion masks mapped to the left hemisphere of the template cortical surface. The distribution of FCDs across the cerebral cortex is nonuniform, with higher concentrations in the superior frontal sulcus, frontal pole, temporal pole, and superior temporal gyrus. (B) Three‐dimensional lesion likelihood atlas. Aggregated surface‐based lesion map values were normalized to between 0 and 1 and mapped back to the template magnetic resonance imaging volume. (C) Sample size required for consistent FCD lesion map. Rank correlation (y axis) was calculated by comparing the lesion map from a smaller cohort to a larger withheld cohort (*n* = 250). *r_rank_* increased with sample size. Predictive learning curve (red line) estimated that a stable map of lesion distribution requires a sample size of *n* = 400. (D) Distribution of FCD lesions according to histopathological subtype. (E) Distributions of lesions across cortical lobes within each FCD histopathological subtype. The width of bars indicates the relative numbers of patients. Temporal lobe lesions made up larger proportions of FCD Types I and III, whereas frontal lobe lesions were more likely to be FCD Types IIA and IIB

Five hundred forty‐eight patients had lesion masks (T1‐weighted MRI; 1.5 T, *n* = 98; 3 T, *n* = 450; FreeSurfer v5.3, *n* = 401; v6, *n* = 147). The 32 patients who did not have lesion masks were excluded from subsequent analyses. FCDs were evenly distributed between left (L) and right (R) hemispheres (L:R = 266:282). Lesions were located throughout the cortex (262 frontal, 158 temporal, 90 parietal, 20 occipital, 10 cingulate, eight insula). The MRI scans of 188 patients (32%) had at some point been reported as MRI‐negative, with a similar proportion of patients scanned on 1.5‐T or 3‐T MRI being reported as MRI‐negative. Lesions were nonuniformly distributed, with propensity for FCDs in the superior frontal sulcus, frontal pole, temporal pole, and superior temporal gyrus (Figure 1A,B). Lesion maps were unstable at sample sizes typical for neuroimaging cohorts of FCD, but improved as sample size increased (Figure 1C). A predictive learning curve determined that a sample size of around *n* = 400 would be required for a stable distribution of lesions across the cortex. This provided support that our cohort (*n* = 548) is sufficient to be representative.

Lesion maps of histopathological subtypes (Figure 1D) showed that the distribution of FCD lesions across the cortex differs according to histopathological subtype, with a greater proportion of FCD Type I and III lesions located in the temporal lobe (Figure 1E). In nonoperated patients, lesions were most frequently located in the left inferior frontal gyrus and bilateral insula (Figure 1D, Supplementary Figure S2). Although numbers in this cohort were relatively small, this may represent deliberate avoidance of surgery in language areas and/or challenges in the diagnosis or surgical planning of insula lesions.

### 3.3 Relationships between demographic variables and lesion distribution

**FIGURE 2:**
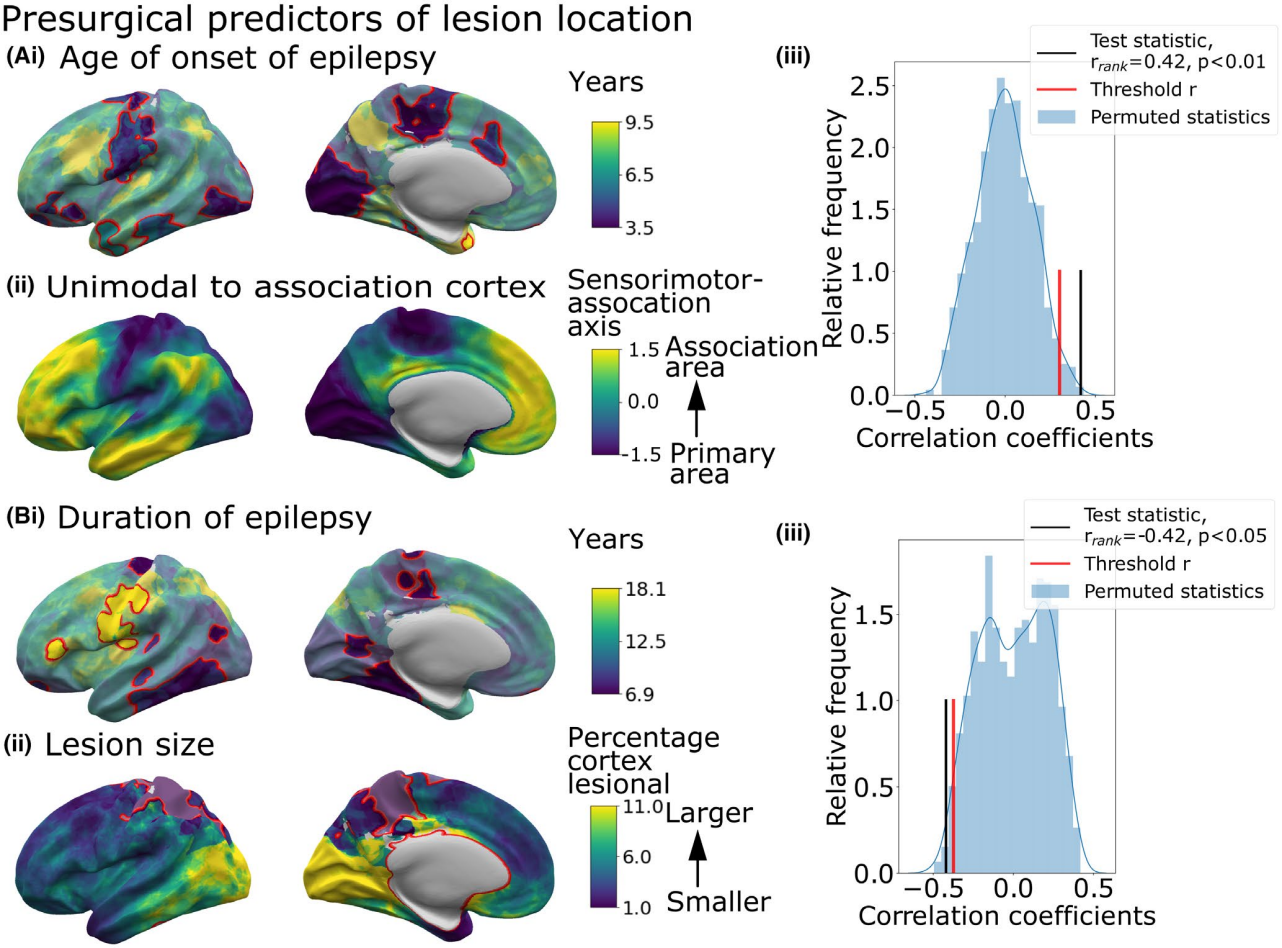
Presurgical factors associated with lesion location. Surface‐based maps show distribution of demographic variables according to lesion location. The color at each vertex represents the average variable value for patients with overlapping lesions. Vertices where the presence of a focal lesion was significantly associated with that variable are delineated in red. Factors significantly (*p* < .01) associated with lesion location were (Ai) age at epilepsy onset, (Bi) duration of epilepsy, and (Bii) lesion size. (Aiii) Correlation between the sensorimotor‐association axis of cortical organization (Aii) and the age at epilepsy onset (Ai) maps in comparison to spatially permuted maps. Lesions in primary areas were associated with a younger age at onset, whereas association areas had older ages of onset (*r_rank_* = 0.39, *p_spin_* < .01). (Biii) Correlation between the duration of epilepsy (Bi) and lesion size (Bii) maps in comparison to spatially permuted maps. Mean duration was significantly negatively correlated with the size of epilepsy lesion, where cortical areas with smaller lesions, for example, precentral and frontal areas, were associated with a longer duration of epilepsy, whereas areas with larger lesions, for example, occipital cortex, had shorter durations of epilepsy (*r_rank_* = −0.42, *p_spin_* < .05)

Age at epilepsy onset, duration of epilepsy, and lesion size were significantly related to lesion location (number of significant vertices > expected by chance, *p *< .01; Figure 2). Hemisphere, sex, and ever reported MRI‐negative were not. The age at onset map (Figure 2A) was significantly correlated with the principal axis of cortical developmental organization. Lesions in primary areas were associated with a younger age at onset, whereas association areas had older ages at onset (*r_rank_* = 0.42, *p_spin_* < .001). Overall, lesions in temporal, parietal, and occipital cortices were associated with a shorter duration of epilepsy (Figure 2B), whereas lesions around the central sulcus and frontal lobe were associated with a longer duration of epilepsy. This pattern closely resembled the distribution of lesion size and age at scan (Figure 2C,D), whereby cortical areas associated with longer durations like the frontal lobe tended to have smaller lesions (*r_rank_* = −0.42, *p_spin_* < .05) and patients tended to have been older at MRI scan (*r_rank_* = 0.92, *p_spin_* < .001).

### 3.4 Factors associated with seizure freedom

**FIGURE 3:**
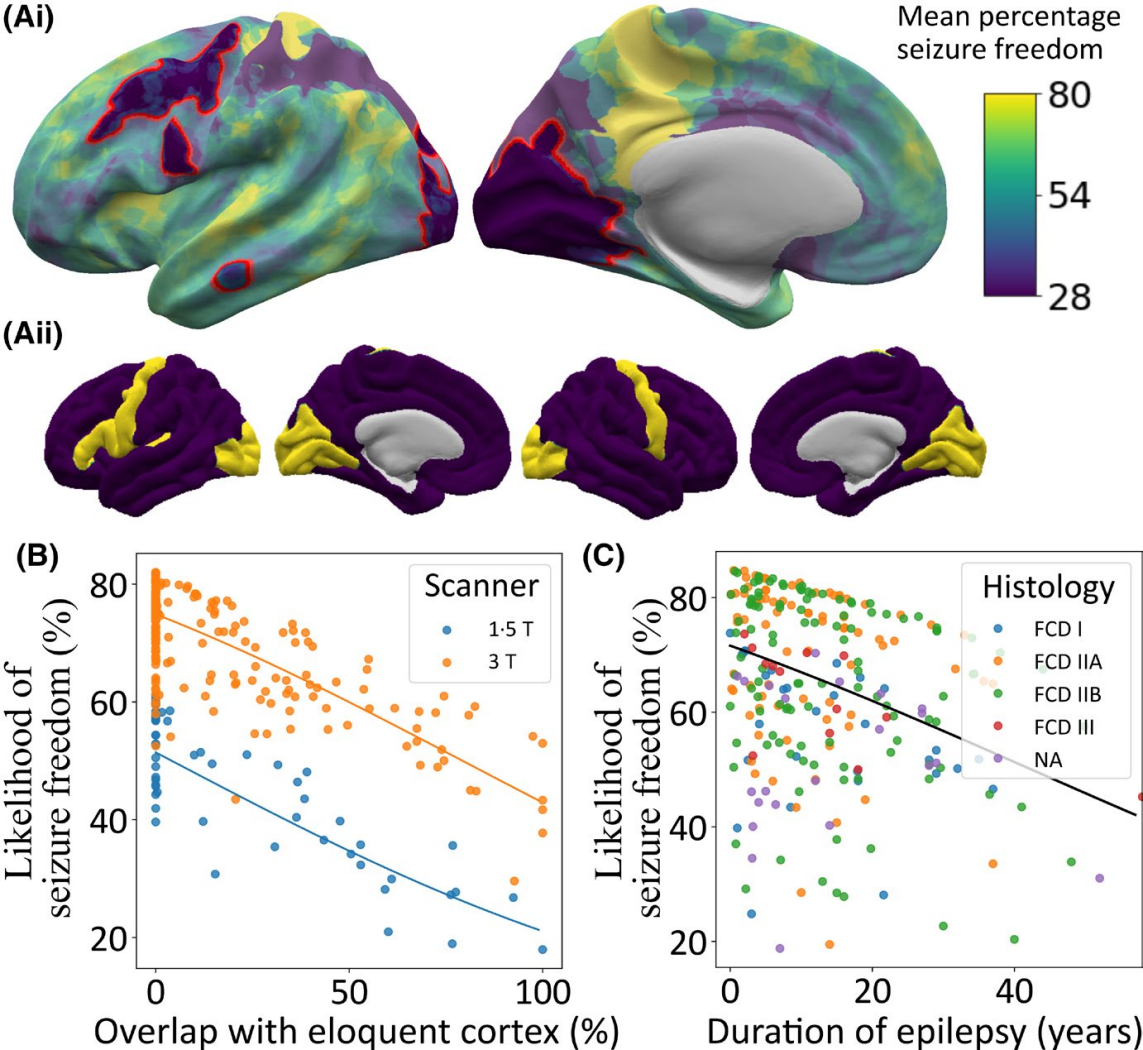
Effect of lesion location, duration of epilepsy, and histopathological subtype on seizure freedom. (Ai) Percentage of patients seizure‐free (%) according to lesion location across the cerebral cortex. Visual, motor, and premotor areas had a low percentage of seizure‐free patients (30%–40%). (Aii) Mask of eloquent cortex. (B) Impact of overlap of lesion with eloquent cortex and magnetic resonance imaging scanner field strength on likelihood of seizure freedom. (C) Impact of duration and histopathology on predicted percentage likelihood of seizure freedom. FCD, focal cortical dysplasia; NA, not available

The percentage of patients who were seizure‐free postsurgery varied according to lesion location (Figure 3A). The first logistic regression model, based solely on vertexwise lesion location, showed that visual, motor, and premotor areas were associated with significantly lower seizure freedom rates (30%–40% of patients were seizure‐free), likely reflecting conservative resection around eloquent cortex.

The second model was fitted with duration of epilepsy, age at epilepsy onset, MRI‐negative status, scanner, lesion overlap with eloquent cortex, and lesion size. Seizure freedom decreased as the overlap with eloquent cortex increased (Figure 3B; *β* = −1.39, *p* < .001), with a 50% overlap between the lesion and eloquent cortex associated with a 16% decrease in the likelihood of seizure freedom compared to no overlap. Being scanned on a 1.5‐T MRI scanner was associated with lower likelihood of seizure freedom, approximately 25% lower, but may be confounded by intersite and temporal trends (Figure 3B; *β* = 1.04, *p* < .001). Likelihood of seizure freedom decreased with longer duration of epilepsy (Figure 3C; *β* = −1.23, *p* < .01), with a 10‐year increase in duration of epilepsy associated with a 5% decrease in likelihood of seizure freedom. There was no significant association between age at epilepsy onset, lesion size, or MRI‐negative status and postsurgical seizure freedom. However, the directions of the effects were in keeping with previous literature, where larger lesions (*β* = −0.66) and having been reported MRI‐negative (*β* = −0.32) tended toward worse outcomes. In the separate model, including age at MRI scan, seizure freedom decreased as age at MRI scan increased (*β* = −1.07, *p* < .01).

The third model additionally included histopathological diagnosis and an interaction term between histopathological diagnosis and lesion size. There was no significant association between FCD subtype and seizure freedom, nor was there an interaction between FCD subtype, lesion size, and seizure freedom. Duration of epilepsy, scanner field strength, and overlap with eloquent cortex remained significant.

Tenfold cross‐validation of the second model including only presurgical variables was used to calculate the predictive value of the model. The model for predicting seizure freedom had a sensitivity of 92%, specificity of 30%, positive predictive value of 70%, and negative predictive value of 67%.

### 3.5 Interrelationships between demographic, lesional, and surgical variables

**FIGURE 4:**
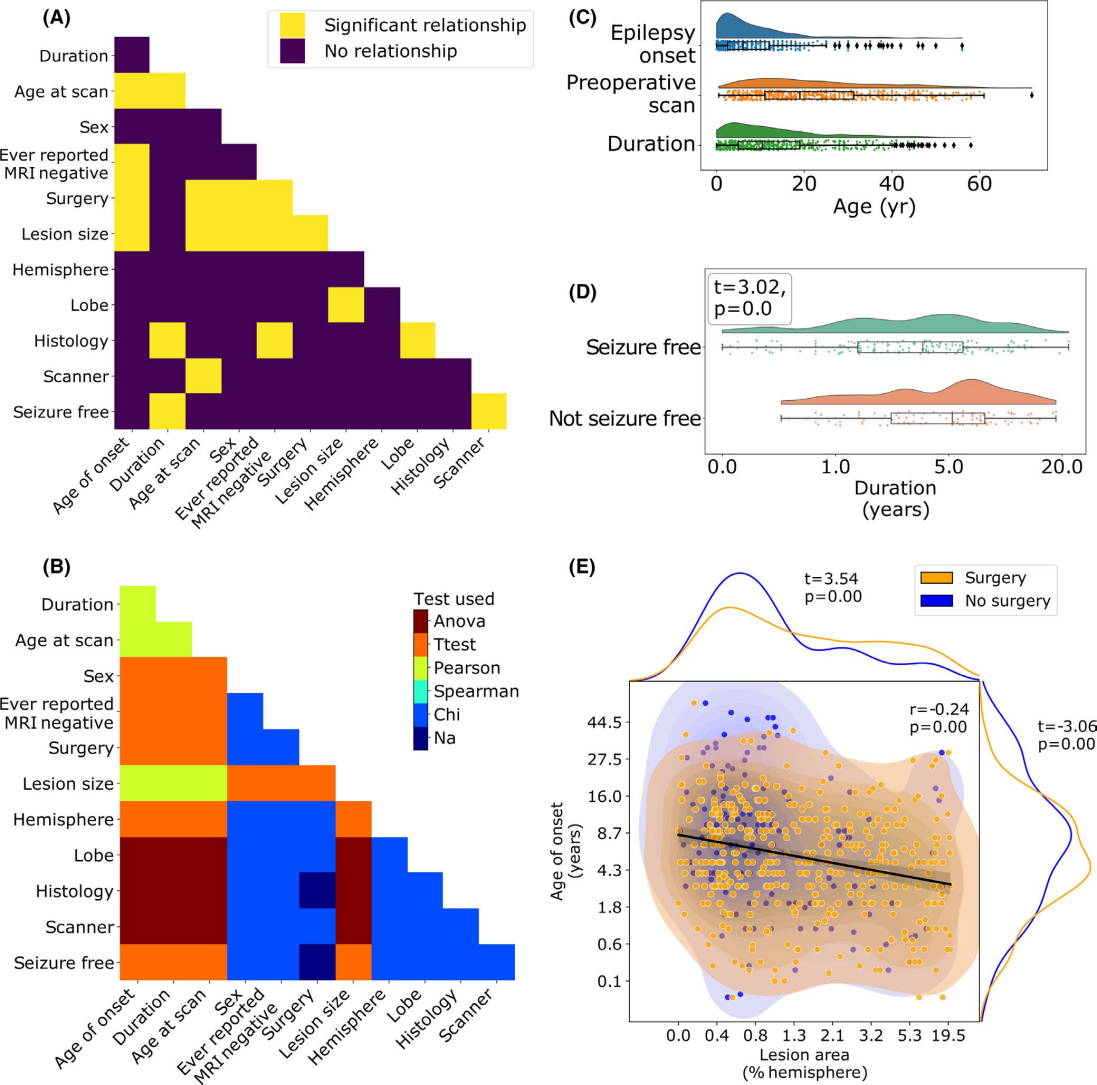
Interrelationships between features. (A) Pairwise comparison of demographic and clinical features. Significant relationships after correction for multiple comparisons are shown in yellow. (B) Statistical test used for each pairwise comparison. (C) Distributions of age at epilepsy onset, age at magnetic resonance imaging (MRI) scan, and duration of epilepsy. (D) Duration of epilepsy is significantly associated with seizure freedom (*t* = −3.0, *p* < .001). Patients with longer durations of epilepsy are less likely to be seizure‐free. (E) Age at epilepsy onset, lesion size (as a percentage of the total hemisphere size), and seizure freedom are significantly associated. Larger lesions are associated with younger age at epilepsy onset (*r* = −0.24, *p* < .001) and are more likely to be operated on (*t* = 3.69, *p* < .001). Similarly, patients with a younger age at epilepsy onset are more likely to be operated on (*t* = −3.76, *p* < .001). Anova, analysis of variance; Na, not applicable

Figure 4A displays significant relationships between demographic, lesional, and surgical variables after systematic evaluation of all interrelationships. Full statistics are reported in Supplementary Figure S3. Results of interest are highlighted below.

### 3.6 Relationships with age at MRI scan

The distributions of age at epilepsy onset, age at MRI scan, and duration of epilepsy revealed that overall there was a long interval between patients developing epilepsy and having their MRI scan (Table 1, Figure 4C). Although 68% of patients have epilepsy onset before the age of 10 years, 51% of patients wait >10 years before having their MRI scan and consequently undergoing presurgical evaluation. There was a small but significant negative correlation between age at scan and lesion surface area (*r* = −0.19, *p* < .001), that is, patients with larger lesions had presurgical evaluation at a younger age. Patients scanned with 1.5‐T MRI were on average younger than those with 3‐T MRI (*F* = 8.58, *p* < .001). Lastly, older patients were less likely to have had surgery (*t* = −3.76, *p* < .001).

### 3.7 Relationships between lesion size, histopathology, surgery, and ever reported MRI‐negative

Patients with earlier epilepsy onset had larger lesions (*r* = −0.24, *p* < .001; Figure 4E), were less likely to have ever been reported MRI‐negative (*t* = −3.70, *p* < .001), and were more likely to have had epilepsy surgery (*t* = −3.38, *p* < .01; Figure 4E). Patients with larger lesions were more likely to have had surgery (*t* = 3.69, *p* < .001; Figure 4E). FCD Type IIA patients were more likely to have been MRI‐negative than FCD Type IIB patients (*χ* ^2^ = 12.2, *p* < .01, Tukey post hoc, *p* < .01).

### 3.8 Relationships with lesional lobe

Lesion surface area was significantly associated with lobe (*F* = 6.9, *p* < .001), driven by temporal lobe lesions being larger than frontal (Tukey post hoc *p* < .01) and parietal lesions (Tukey post hoc *p* < .05). FCD Type I and III lesions were more frequently located in the temporal lobe (*χ^2^* = 70.2, *p* < .001, Tukey post hoc *p* < .001; Figure 1E).

### 3.9 Relationships with seizure freedom

Patients with longer durations of epilepsy were less likely to be seizure‐free (*t* = −3.0, *p* < .001). Patients with 1.5‐T MRI scans were less likely to be seizure‐free than those with 3‐T imaging (*χ* ^2^ = 15.7, *p* < .001). No other factors survived correction for multiple comparisons (Supplementary Figure S3).

## 4 DISCUSSION

In this multicenter study of 580 patients with FCD, lesions were nonuniformly distributed across the cerebral cortex, with predominance in the superior frontal sulcus, frontal pole, and temporal pole. Lesion location was significantly associated with age at epilepsy onset, duration of epilepsy, age at MRI scan, and lesion size. Likelihood of seizure freedom postoperatively varied considerably according to lesion location, with lesions in visual, motor, and premotor areas associated with much lower rates of seizure freedom than elsewhere, likely attributable to neurosurgical caution in resecting lesions around eloquent cortex. A model incorporating overlap with eloquent cortex alongside duration of epilepsy, age at epilepsy onset, MRI scanner field strength, whether the patient was ever reported MRI‐negative, and lesion size had a positive predictive value of 70% and negative predictive value of 67%.

The atlas of FCD lesion location highlighted a nonuniform distribution across and between cortical lobes (Figure 1). It substantiated previous studies finding that FCDs were more common in frontal (particularly FCD Type II) and temporal (FCD Types I and III) lobes.^3^, ^4^ Additionally, this atlas extends previous understanding, demonstrating “hot spots” in the superior frontal sulcus driven by FCD Type IIB, and a cluster of temporal pole lesions in all FCD subtypes. Understanding sites of predilection for FCD focus aids the clinical search for FCDs. Furthermore, regional variations in FCD incidence provide directions for research, including whether the causative somatic mosaic mutations occur nonuniformly^22^ or whether regional variations in cortical molecular expression,^23^ electrophysiology,^24^ laminar organization,^25^ or connectivity^26^ determine lesional epileptogenicity.

Linking individual clinical and demographic data with lesion location uncovered relationships between age at epilepsy onset, duration of epilepsy, lesion size, and lesion location. Lesions in primary sensory, motor, and visual areas were associated with earlier epilepsy onset, whereas lesions in higher order association cortex were associated with later epilepsy onset (Figure 2A,E). This may reflect differing developmental trajectories of these areas,^19^, ^27^, ^28^ with seizures initiating as a result of the maturation of particular cortical properties first in primary areas. However, differing seizure semiologies may also be a contributing factor, where more subtle seizure symptoms are not attributed to a diagnosis of epilepsy of longer duration.

Most patients had epilepsy onset during childhood (median age at onset = 6.0 years), but the median age at MRI scan was 19.0 years (Table 1), indicating many patients had long delays between epilepsy onset and potentially curative epilepsy surgery (Figure 4C; median duration = 10.4 years). Longer duration of epilepsy is associated with increased morbidity and mortality, and with worse postsurgical outcome (Figures 3B and 4A).^4^, ^29^ In our cohort, patients with a longer duration of epilepsy were more likely to have lesions in the frontal cortex, particularly around the central sulcus. Factors contributing to longer duration of epilepsy might include diagnostic and surgical challenges such as lesion conspicuity, MRI scanning protocol, and whether a lesion is in eloquent cortex. In keeping with this, larger lesions were more likely to have been operated on (Figure 4D). Other reasons for prolonged durations of epilepsy might include trials of antiepileptic drugs, alongside underreferral and delayed referral to specialist epilepsy centers.^30^

Consistent with a recent study,^4^ 66% of patients with FCD in the MELD cohort were seizure‐free postsurgically. We found that a longer duration of epilepsy was significantly associated with a reduced chance of seizure freedom (Figure 4A), but the impact of duration was small, with a 5% decrease in likelihood of seizure freedom for every 10‐year increase in duration of epilepsy (Figure 3C). By contrast, there was a striking effect of lesion location on seizure freedom, with the likelihood of seizure freedom dropping from 70% when lesions had no overlap with eloquent areas to 54% when there was 50% overlap, representing a 16% decrease (Figure 3B). The most likely reason for this is that neurosurgical resection plans were intentionally cautious in an attempt to avoid resecting motor and visual cortex or the adjacent white matter tracts to minimize the risk of deficits such as hemiparesis or hemianopia,^31^ leading to higher rates of incomplete resection.^11^

The predictive model of postoperative seizure freedom, which included overlap with eloquent cortex, duration of epilepsy, age at epilepsy onset, MRI scanner field strength, whether the patient was ever reported MRI‐negative, and lesion size, had a sensitivity of 92% and demonstrated predictive power on unseen data. However, the specificity of the model was only 30%, indicating that many patients who continue to have seizures are predicted to have a likelihood of seizure freedom of >50%. Poor outcomes in these patients are likely to be due to surgical factors, such as incomplete resection, and electrophysiological characteristics of the patients, that is, the likelihood of multiple epileptogenic sources, which are not included in the model. Models integrating the factors included in this model with other established clinical predictors^32^, ^33^ may improve predictive modeling of seizure freedom and provide a useful aid in presurgical decision‐making.

One strength of our study is the inclusion of both operated and nonoperated patients. This helped to minimize ascertainment bias in the dataset, as the lesional distribution of nonoperated patients captured more lesions in eloquent language areas (e.g., left inferior frontal gyrus) or cortex which/that is more surgically challenging to resect (e.g., insula; Supplementary Figure S2), whereas purely postsurgical cohorts may undersample these patients. However, it is important to note that our cohort primarily consisted of patients with epileptogenic, drug‐resistant FCDs from epilepsy surgery centers. Independent studies are needed to establish the distributions of nonepileptogenic or drug‐responsive FCDs, which may not present to such centers.

One limitation is the number of clinical and MRI variables collected. Future work including more detailed clinical information, such as seizure types, seizure burden, electrophysiology, medication, genetics, and extent of lesion resection from postoperative MRI scans might further our understanding of FCD. In particular, identifying patients with incomplete resections will improve the low specificity of the model predicting likelihood of seizure freedom. Additionally, the clinical variable “ever reported MRI‐negative” is imperfect, depending on MRI protocols and the expertise of radiological review. Nevertheless, over a large sample size, it does indicate that some lesions were more subtle.

As a subtle pathology on MRI, manual masking of FCD lesions is challenging. There is likely to be some heterogeneity in the masking process. Lesions masked based on visually identified features on the T1 image may underestimate the dysplastic tissue, whereas MRI‐negative lesions masked based on resection cavities may overestimate lesion extent. Future studies using automated lesion detection and masking^7^ may yield more observer‐independent and consistent lesion masks. Nevertheless, the stability of the lesion distribution pattern when randomly subsampling the cohort (Figure 1C) indicates that the core findings of this study are robust to idiosyncrasies of particular lesion masks.

Large collaborative initiatives have shown the power of big data to answer clinically relevant questions.^9^, ^34^, ^35^ Here, open‐science practices enabled mapping of the topographic distribution of epileptogenic FCDs across the cerebral cortex, a departure from the coarse, lobar annotations usually described. The surface and volumetric atlas of lesion location will serve as a diagnostic and teaching aid in the radiological search for an individual patient's subtle lesion. The predictive models of postsurgical seizure freedom can be used to estimate individual postsurgical seizure freedom based on a lesion's proximity to eloquent cortex. This could also inform presurgical decision‐making, resection planning, and risk counseling of patients. Lastly, through making all our code available and providing user‐friendly interactive notebooks detailing how to run all analyses, this framework can be replicated by researchers in epilepsy and other focal neurological conditions.

## CONFLICT OF INTEREST

None of the authors has any conflict of interest to disclose.

## Supporting information

Fig S1–S3
