# Supplementary material for: Atlas of lesion locations and postsurgical seizure freedom in focal cortical dysplasia: A MELD study
Source: Epilepsia. 2021 Nov 29;63(1):61–74. doi: 10.1111/epi.17130 (PMC8916105; doi:10.1111/epi.17130)
Supplement: Supplementary file 1 — Fig S1–S3 [file EPI-63-61-s001.docx]

**
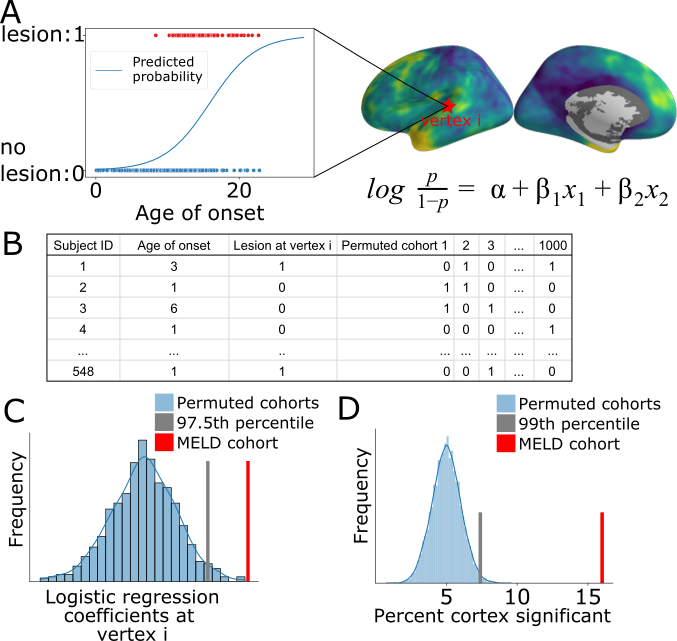
**

**Supplementary Figure 1. Overview of logistic regression framework to identify presurgical predictors of lesion location.** (**A**) Logistic regression model including age of epilepsy onset, sex, ever reported MRI-negative, duration of epilepsy and lesion hemisphere was fitted to predict the presence / absence of a lesion at every vertex. (**B**) 1000 permuted cohorts created where presence / absence of a lesion at a particular vertex is random. (**C**) Normal distribution of coefficients from permuted cohorts at vertex i. The coefficient from actual data (red) is greater than 97.5% of the coefficients from the permuted cohorts, and therefore vertex i is considered significant. (**D**) Normal distribution of the percentage of vertices that were significant for a particular factor (e.g., age of onset) in the permuted cohorts. The factor (red) was considered significantly related to lesion location as the number of significant vertices exceeded the 99th percentile value from the permuted cohorts.


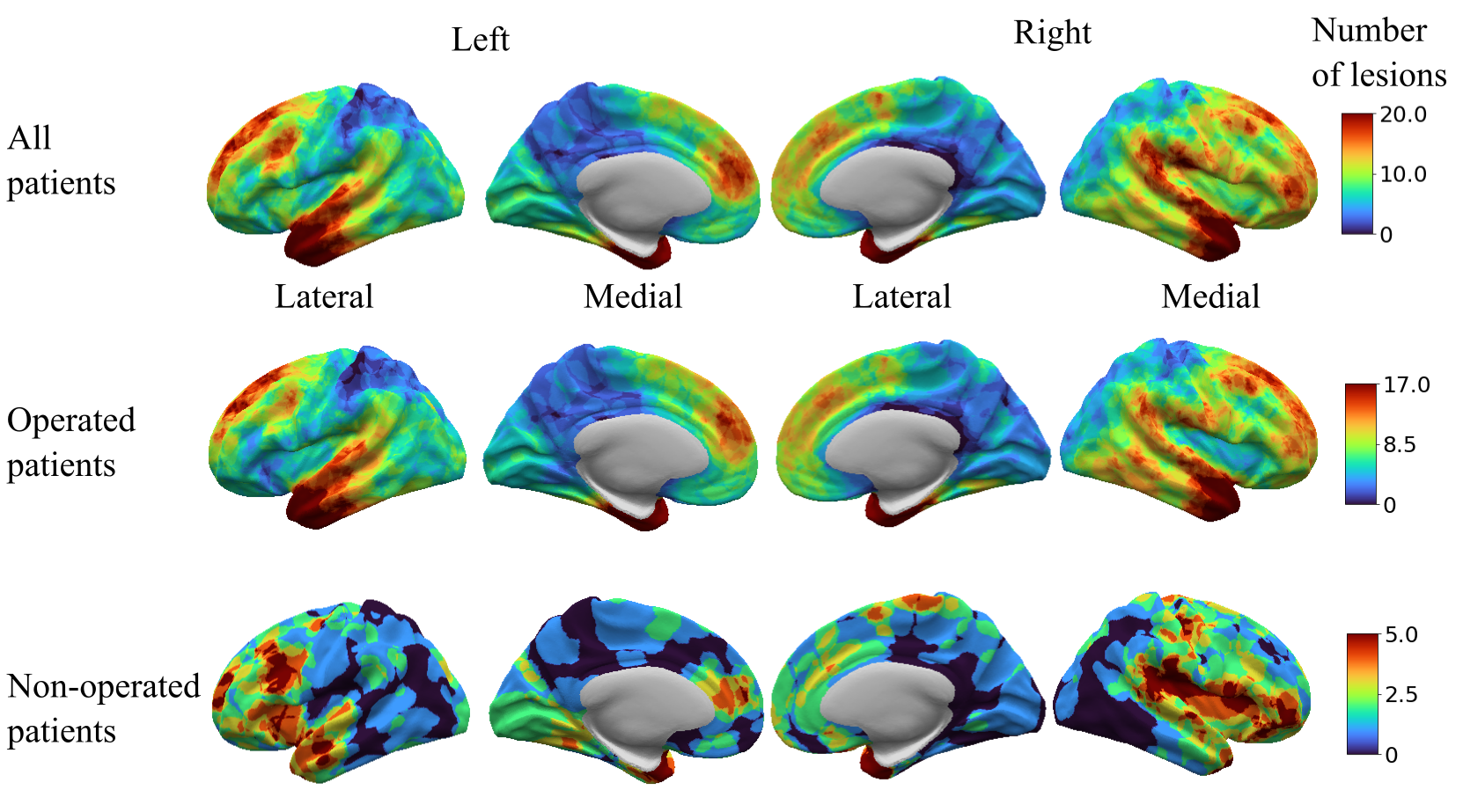


**Supplementary Figure 2. Bilateral distributions of FCDs.** Distributions of FCD lesions on left and right hemispheres in the whole MELD cohort, patients who had undergone surgery and those that had not. The pattern in the whole and operated cohorts appear symmetric, whereas patients who had not undergone surgery appeared to have more lesions in the left inferior frontal gyrus, near Broca’s area. Additionally lesions in the non-operated cohort appear to be more frequently located in the insula, a diagnostically and surgically challenging area for cortical resection.


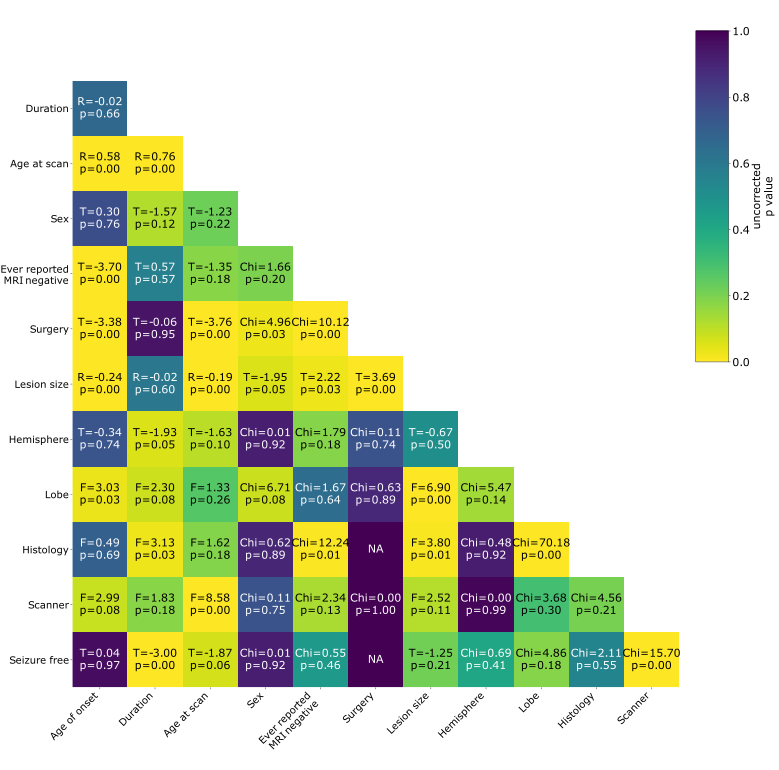


**Supplementary Figure 3. Pairwise comparisons between all demographic and clinical features**. Test statistics and uncorrected p values are displayed. See Figure 4 for a more detailed exploration of findings.
